# *Hc-hrg-2*, a glutathione transferase gene, regulates heme homeostasis in the blood-feeding parasitic nematode *Haemonchus contortus*

**DOI:** 10.1186/s13071-020-3911-z

**Published:** 2020-01-29

**Authors:** Jing-Ru Zhou, Dan-Ru Bu, Xian-Feng Zhao, Fei Wu, Xue-Qiu Chen, Heng-Zhi Shi, Chao-Qun Yao, Ai-Fang Du, Yi Yang

**Affiliations:** 1 Institute of Preventive Veterinary Medicine, Zhejiang Provincial Key Laboratory of Preventive Veterinary Medicine, College of Animal Sciences, Zhejiang University, Hangzhou, 310058 People’s Republic of China; 2 Shenzhen Entry-exit Inspection and Quarantine Bureau, Shenzhen, Guangdong 518045 People’s Republic of China; 3 Ross University School of Veterinary Medicine and One Health Center for Zoonoses and Tropical Veterinary Medicine, Ross University School of Veterinary Medicine, P.O. Box 334, Basseterre, Saint Kitts and Nevis

**Keywords:** *Haemonchus contortus*, Heme, *Hc-hrg-2*, Glutathione S-transferase

## Abstract

**Background:**

*Haemonchus contortus*, a blood-feeding parasite, is constantly surrounded by large quantities of heme released from the catabolism of host red blood cells. To cope with the toxicity of free heme, *H. contortus* needs to uptake and detoxify the heme, a process believed to be paramount for parasite survival.

**Methods:**

A heme-responsive gene *Hc-hrg-2* was identified which is the homologue of *Ce-hrg-2.* The transcriptional levels in all developmental stages and heme-responsive ability of *Hc-hrg-2* were analyzed by qRT-PCR. Immunofluorescence analysis and cell transfections were performed to analyze the expression pattern of Hc-HGR-2. Statistical analyses were performed with GraghPad Prism 6.0 using Student’s t-test.

**Results:**

To investigate the heme homeostasis of *H. contortus*, we first identified a heme-responsive gene *Hc-hrg-2*, a homolog of *Ce-hrg-2* that is involved in heme transport in the hypodermis of *Caenorhabditis elegans*. Using qRT-PCR, we showed that *Hc-hrg-2* mRNA was expressed throughout all life-cycle stages of *H. contortus* with the highest level in the third-stage larvae (L3s). Notably, transcription of *Hc-hrg-2* in the exsheathed L3s was significantly upregulated in the presence of high concentration of heme. We found that Hc-HRG-2 protein was mainly located in the hypodermal tissues of adult *H. contortus in vivo* and the endoplasmic reticulum in the transfected mammalian cells. Our *in vitro* assay demonstrated that Hc-HRG-2 is a heme-binding protein with glutathione S-transferase activity and heme had a significant effect on its enzymatic activity when a model substrate 1-chloro-2, 4-dinitrobenzene (CDNB) was used.

**Conclusions:**

*Hc-hrg-2* is a heme-responsive gene and engaged in heme homeostasis regulation in hypodermal tissues during the free-living stages of *H. contortus*.
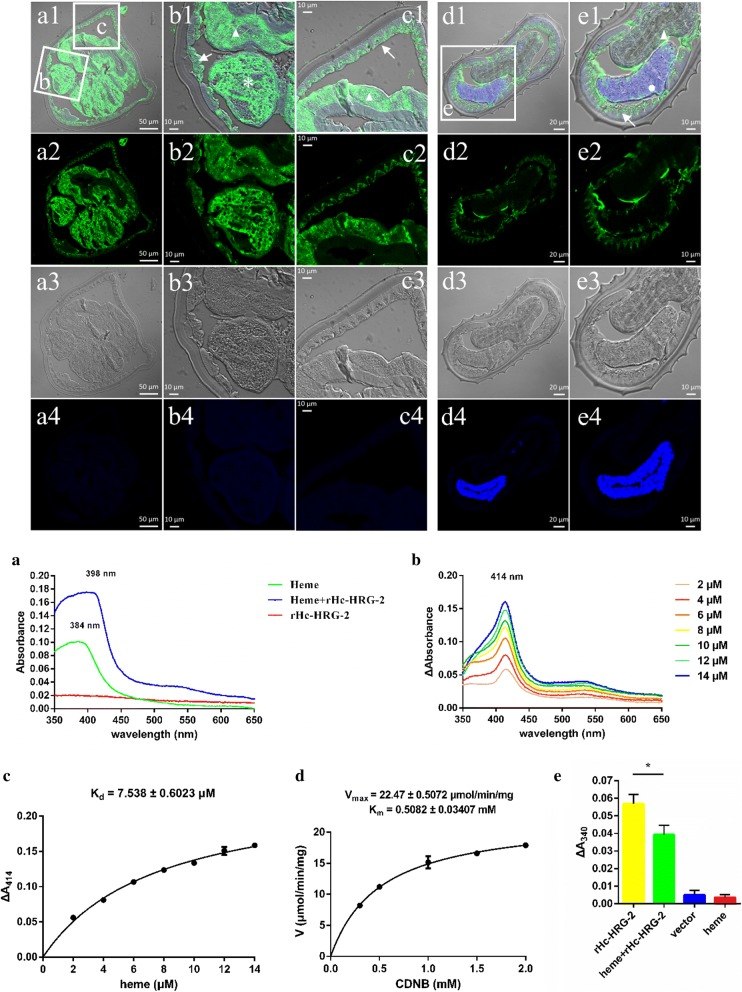

## Background

*Haemonchus contortus*, a gastrointestinal blood-feeding nematode, poses a major economic burden to agricultural communities worldwide. Its infection leads to anemia, weight loss and ultimate death of the small ruminants [1]. Currently, the most effective control method for the prevention of *H. contortus* infection is the rational combination of pasture management and anthelmintic treatment [2]. However, frequent treatment enhances the development of anthelmintic resistance of the parasite [3]. It is thus increasingly urgent to identify essential molecular pathways of parasite which can be used to find potential new drug targets [4].

Heme is an iron-containing porphyrin and acts as a cofactor for proteins involved in a variety of cellular functions, including oxygen transport, electron transfer and cell signaling [5, 6]. However, despite its necessity, free heme can generate both hydroxyl radicals and reactive oxygen species (ROS), which are potentially toxic and capable of damaging and degrading proteins, lipids and DNAs [7–9]. Therefore, cells must be able to carefully regulate, compartmentalize and transport heme to target hemoproteins [10].

As most heme in a living body is contained within the red blood cells (RBC) loaded with hemoglobin [11, 12], the blood-feeding parasites, such as *H. contortus*, constantly cope with a mass of heme, released from their catabolism of host RBC [13]. A hemoglobin-like protein and cathepsin L-like cysteine protease involved in hydrolyzing hemoglobin (Hb) have been found in the L4-stage and adult *H. contortus* [14, 15]. This, along with the fact that *H. contortus* cannot synthesize heme *de novo* [13], suggests that this heme auxotrophy must have both an intercellular heme transport system to mobilize heme from intestinal cells where heme is absorbed to other cell types including neurons, muscles, hypodermis, and a heme detoxification mechanism required to excrete and detoxify the bulk of ingested heme [10, 13].

Multiple heme transporters have been first identified in the free-living model organism *Caenorhabditis elegans* and named HRG (heme-responsive genes) [10, 16]. Studies on the mechanisms for heme uptake in protozoans, such as *Trypanosoma brucei* and *Leishmania amazonensis*, have shown a strong positive correlation between heme availability and parasite growth, survival and reproduction [17, 18]. Evidence for uptake of heme or heme analogs have also been found in the trematode *Schistosoma mansoni*, the cattle tick *Boophilus microplus* and the nematode *Nippostrongylus brasiliensis* [19–21]. It is worth noting that multiple heme-responsive GSTs with high affinity binding to heme have been identified in *H. contortus* and *Ancylostoma caninum* indicating that GST is important for transport and detoxification of heme [2, 22]

In this study, we first identified a heme-responsive gene *Hc-hrg-2*, a homology of *Ce-hrg-2* which is involved in heme transport in the hypodermis of *C. elegans* [23]. We described the expression pattern and carried out a functional study of this newly identified gene, with the aim of investigating its role in regulating the heme utilization in *H. contortus.*

## Methods

### Animals and parasites

Sheep, under helminth-free conditions, were infected intragastrically with 8000 infective third-stage larvae (iL3s) of *H. contortus* Zhejiang (ZJ) strain. Eggs in the feces were detected at day 21 post-infection using the floating method [24]. Briefly, sheep feces mixed with saturated saline were centrifuged at 8000× *g* for 5 min. Materials in the top layer filtrate were passed through sequential nylon screens of various pore sizes to remove fine debris and the eggs of *H. contortus* were collected on a 37 μm nylon mesh screen. The first-stage larvae (L1s), second-stage larvae (L2s) and iL3s were collected from eggs incubated for 1, 3 and 7 days, respectively, in fecal culture medium at 28 °C [25, 26]. To get rid of the fecal debris, larvae were collected from fecal culture medium and then passed through a 23 μm nylon mesh screen overnight. The fourth-stage larvae (L4s) and adults were collected from sheep abomasa and washed in phosphate-buffered saline (PBS). All samples were stored in liquid nitrogen until use.

### Isolation of full-length cDNA and genomic DNA of *Hc-hrg-2* from *H. contortus*

The total RNA of worms at different developmental stages or exposed to different concentrations of heme were extracted using Trizol reagent (Invitrogen, Carlsbad, CA, USA). The first strand cDNA for the conventional PCR amplification was obtained using the First Strand cDNA Synthesis Kit (Toyobo Co., Ltd., Osaka, Japan) by following the manufacturerʼs protocol. For real-time PCR, the first strand cDNA was obtained by ReverTra Ace qPCR RT Kit (Toyobo Co., Ltd.). The genomic DNA was extracted from adult worms using a TIANamp genomic DNA kit (Tiangen Biotech Co., Ltd., Beijing, China). All samples were stored at − 80 °C until use.

The translated amino acid of *Ce-hrg-2* gene (WormBase ID: WBGene00010473) was used to search the *H. contortus* genomic database (https://blast.ncbi.nlm.nih.gov/Blast.cgi). The sequence, numbered CDJ97722.1, shared high homology with *Ce-hrg-2* and was identified and named as *H. contortus* heme-responsive gene-2 (*Hc-hrg-2*) (GenBank: MK371241). Full-length cDNA was synthesized from total RNA of *H. contortus* adults using gene-specific primer pair amplification and 3’ RACE kit (TaKaRa, Dalian, China) (Table 1). The full-length genomic DNA of *Hc-hrg-2* was obtained from the sequencing PCR products that were derived in PCR amplification using primers designed based on the acquired cDNA sequence (Table 1) and cloned into pMD18-T vectors (TaKaRa). The complete cDNA and genomic DNA sequences were used to determine intron/exon boundaries.

**Table 1:** Primers used in this study

Primer ID	Used for	Primer sequence (5ʹ–3ʹ)
*Hc-hrg-2-*F	cDNA and genomic DNA	ATGATTCTCTTGGTTTCTGTTGCTG
*Hc-hrg-2-*R	cDNA and genomic DNA	AGCAAACTCTTTTCCAAAAACCGTATCTCG
PE*-Hc-hrg-2-*F	Prokaryotic expression	CGGGATCCATGATTCTCTTGGTTTCTGTTGCTG
PE*-Hc-hrg-2-*R	Prokaryotic expression	CCCAAGCTTTCATTCTTCAGCAAACTCTTTTCCAAAAA
*RA-Hc-hrg-2-*R1	3ʹ RACE	CCTAAATTACCTGTCCCACTCCC
RA*-Hc-hrg-2-R2*	3ʹ RACE	GTTAAAACGAGCGTGAGGCCAT
real*-Hc-hrg-2-*F	real-time PCR	ACTGCCCATAGTGCTTCCAC
real*-Hc-hrg-2-*R	real-time PCR	AGTCCTCGACAGGGAACTGA
Real*-Hc-actin-1-*F	real-time PCR	TCAATTGTCGGACGTCCTCG
Real*-Hc-actin-1-*R	real-time PCR	AGGGGAGCTTCGGTCAAAAG
EE-*Hc-hrg-2*-F	Eukaryotic expression	CGGAATTCATGATTCTCTTGGTTTCTGTTGCTG
EE-*Hc-hrg-2*-R	Eukaryotic expression	CGGGATCCTCATTCTTCAGCAAACTCTTTTCCAAAAA
EE-*Hc-hrg-2*(ΔTMD)-F	Eukaryotic expression	CGGAATTCATGATTCTCAAGAAGGACAAGAAAGAGGTT
EE-*Hc-hrg-2*(ΔTMD)-R	Eukaryotic expression	CGGGATCCTCATTCTTCAGCAAACTCTTTTCCA
EE-*Hc-hrg-2*(ΔGST-N)-F1	Eukaryotic expression	CGGAATTCATGATTCTCTTGGTTTCTGTTGCTG
EE-*Hc-hrg-2*(ΔGST-N)-R1	Eukaryotic expression	CGACGCGAGAGGCTTCACGGTATCGGATTTCCA
EE-*Hc-hrg-2*(ΔGST-N)-F2	Eukaryotic expression	GTGAAGCCTCTCGCGTCGTCTA
EE-*Hc-hrg-2*(ΔGST-N)-R2	Eukaryotic expression	CGGGATCCTCATTCTTCAGCAAACTCTTTTCCA
EE-*Hc-hrg-2*(ΔGST-C)-F1	Eukaryotic expression	CGGAATTCATGATTCTCTTGGTTTCTGTTGCTGTCTGG
EE-*Hc-hrg-2*(ΔGST-C)-R1	Eukaryotic expression	TTTTCCAAAAACCGTATCCTTGAAGTCCTCGAC
EE-*Hc-hrg-2*(ΔGST-C)-R2	Eukaryotic expression	CGGGATCCTCATTCTTCAGCAAACTCTTTTCCAAAAAC
EE-*Hc-hrg-2*(TMD)-F	Eukaryotic expression	CGACCGGTCGCCACCATGGTGAGCAAGGG
EE-*Hc-hrg-2*(TMD)-R1	Eukaryotic expression	AACGACAGCCAACCAGACAGCAACAGAAACCAAGAAGCTTGAGCTCGAGATCTT
EE-*Hc-hrg-2*(TMD)-R2	Eukaryotic expression	CGGAATTCCTACAAGAAAACAAAATATGCGACGATACTAACGACAGCCAACCAGACAG

*Note*: Restriction sites are underlined

### Sequence analysis

Protein alignment of Hc-HRG-2, Ce-HRG-2 and Hc-GST-1 (GenBank: AF281663) was carried out with Clustal W [27]. The ligand-binding prediction was performed by Iterative Threading ASSEmbly Refinement (I-TASSER) (https://zhanglab.ccmb.med.umich.edu/I-TASSER/) and the transmembrane domain (TMD) was predicted using TMHMM 2.0. The thioredoxin N-terminal domain-like (GST-N) and glutathione S-transferase C-terminal domain-like (GST-C) folds were predicted using InterPro (www.ebi.ac.uk/interpro/). The homologues of Hc-HRG-2 were identified from the data bank of the National Center for Biotechnology Information (https://blast.ncbi.nlm.nih.gov/Blast.cgi) using BLASTp. Phylogenetic analysis of HRG-2 of various nematodes was carried out using the neighbor-joining (NJ) method in MEGA 5.1 with 1000 pseudoreplicates.

### Quantitative reverse transcription-PCR (qRT-PCR) analysis

Quantitative reverse transcription-PCR (qRT-PCR) with specific primer pairs (Table 1) was performed to analyze the relative abundance of *Hc-hrg-2* transcripts in all developmental stages of *H. contortus* (eggs, L1s, L2s, L3s, female L4s, male L4s, female adults and male adults). It was also used to determine transcriptional levels of the gene in the L1s and the exsheathed L3s under various concentrations of exogenous heme (0, 20 or 100 μM).

The qRT-PCR (20 μl system) using SYBR® Green Real-time PCR Master Mix (Toyobo Co., Ltd.) in the CFX96 Touch Real-time PCR System (Bio-Rad Laboratories, Shanghai, China) was performed under the following procedures: 40 cycles of 95 °C for 15 s, 60 °C for 15 s and 72 °C for 30 s. The dissociation curve was generated under the following thermal cycles: 95 °C for 10 s, 65 °C for 5 s and 95 °C for 0.5 s. Each sample was tested in triplicate using actin (*actin-1*) as an internal control. Statistical analysis was conducted using Student’s t-test; *P* ≤ 0.05 was set as the criterion for significance.

### Polyclonal antibody generation

The open reading frame (ORF) of *Hc-hrg-2* was amplified using primers listed in Table 1 with *Bam*HI and *Hind*III restriction sites inserted at the 5ʹ and 3ʹ ends, respectively and ligated into the prokaryotic expression vector pET-32a. The resultant plasmid was then transformed into Rosetta (DE3) cells. The recombinant Hc-HRG-2 (rHc-HRG-2) expression was induced by 1 mM isopropy beta-d-thiogalactopyranoside (IPTG) at 16 °C and the recombinant protein was purified by affinity chromatography using a Ni-NTA agarose column (Qiagen, Shanghai, China), according to the manufacturer’s protocol. The purified protein was used to immunize ICR mice (female, 5-week-old, Shanghai SLAC Laboratory Animal Co., Ltd, China). Briefly, 100 μg/kg purified rHc-HRG-2 was subcutaneously injected into the mice. After 2 weeks, mice were immunized with 50 μg/kg rHc-HRG-2. Ten days later, the immune procedure was finished with injection of 50 μg/kg rHc-HRG-2 into the mice. Seven days after the final immunization, serum was collected from the mice and antibody titer of each serum was determined by enzyme-linked immunosorbent assay (ELISA).

### Mammalian cell transfection

Mammalian cell line HEK293T was maintained in DMEM (Biological Industries, Beit Haemek, Israel) supplemented with 10% (v/v) fetal bovine serum (Biological Industries). *Hc-hrg-2* ORF was amplified with primers (Table 1) flanked by *Eco*RI and *Bam*HI restriction sites. The purified PCR products were cloned into the pmCherry-C2 vector following restriction digestion. Truncated constructs, including HRG-2ΔGST-N, HRG-2ΔGST-C, HRG-2ΔTMD and HRG-2TMD, were individually introduced into mammalian expression plasmids. These DNA constructs and ER marker CD3-EGFP (from Dr Caiyong Chen, College of Life Sciences, Zhejiang University) were transiently transfected into HEK293T cells using Lipofectamine 2000 (Invitrogen) for western blot analysis and fluorescence microscopy studies.

### Western blot analysis

The total protein of *H. contortus* was used to detect the recognition ability of mouse anti-Hc-HRG-2 polyclonal antibody. One hundred mg adult *H. contortus* was well grinded in a glass homogenizer in chilled (4 °C) PBS followed by five freeze-thaw cycles of freezing at -70 °C for 1 h and then thawing at 4 °C for 30 min. The resultant suspension was sonicated using sonicator (Scientz Biotechnology, Ningbo, China) in the presence of 1mM phenylmethanesulfonyl fluoride (PMSF) (Fude Biological Technology, Hangzhou, China) followed by incubation overnight at 4 °C with 10 mM ethylene diamine tetraacetic acid (EDTA) (pH 8.0). The product was then centrifuged at 5000×*g* for 30 min at 4 °C and the supernatant was collected as the PBS-soluble natural protein. The pellet was dissolved in 8 M urea and centrifuged at 8000×*g* for 10 min at 4 °C and the supernatant was collected as the urea-soluble natural protein and stored at − 70 °C until use.

Western bolt was performed to verify (i) the expression of the recombinant Hc-HRG-2 in *Escherichia coli* and HEK293T cells; and (ii) the natural Hc-HRG-2 using the mouse anti-Hc-HRG-2 polyclonal antibodies.

rHc-HRG-2 and the whole cell lysates of transfected HEK293T cells lysed in RIPA lysis buffer (Fude Biological Technology) were separated on a 12% SDS-polyacrylamide gel electrophoresis (SDS-PAGE) and transferred onto a PVDF membrane (Merck KGaA, Darmstadt, Germany). The membrane was blocked in 5% skimmed milk/TPBS (PBS with 0.01% Tween-20) at room temperature for 2 h and subsequently incubated with the primary antibodies at 37 °C for 1 h. Sources and dilution of primary antibodies were as follows: goat anti His-Tag antibody (1:3000, Proteintech Group, Wuhan, China), mouse anti-Hc-HRG-2 polyclonal antibody (1:5000), mouse anti-mCherry monoclonal antibody (1:1000, GeneTex, Taiwan, China) and mouse anti-GFP monoclonal antibody (1:1000, Beyotime Biotechnology, Shanghai, China). Horseradish peroxidase (HRP)-labelled goat anti-mouse IgG (1:8000, Dingguo, Shanghai, China) was used as the secondary antibody, incubated at 37 °C for 1 h. Blots were visualized using FDbio-Femto ECL kit (Fude Biological Technology) under ChemiDoc™ Touch Imaging System (Bio-Rad Laboratories).

### Immunofluorescence analysis

Live adult *H. contortus*, derived from the sheep abomasa of euthanized animals, were fixed in 4% (w/v) paraformaldehyde (Sangon Biotech, Shanghai, China) for 2 days. They were subsequently embedded in paraffin and sectioned to a thickness of 5 μm. The sections were bathed in 100 °C citrate antigen retrieval solution (pH 6.0) for 20 min and then blocked in 10% donkey serum (Absin, Shanghai, China) at 4 °C overnight. Samples were incubated in the primary antibodies (mouse anti-Hc-HRG-2 polyclonal antibody) at a 1:200 dilution followed by fluorescein-conjugated secondary antibody [Alexa FlourTM 488 donkey anti-mouse IgG (H+L) (Invitrogen)] at a 1:500 dilution in the presence of 0.5 μg/ml 4’, 6-diamidino-2-phenylindole (DAPI) (Sigma-Aldrich, Shanghai, China) stain for 20 min. Transfected HEK293T cells grown on cell slides (WHB cientific, Shanghai, China) were fixed with 4% (w/v) paraformaldehyde for 20 min at 4 °C, permeabilized with 0.1% (v/v) Triton X-100 (Sangon Biotech) for 10 min and then stained by DAPI for 30 min. Mounted slides were visualized using a laser scanning confocal microscope (Zeiss LSM 780, Jena, Germany).

### Heme assay

The L1 were sterilized in RPMI 1640 medium (Biological Industries) containing 25 mM HEPES (Solarbio, Beijing, China), 1% fetal bovine serum and an antibiotic-antimycotic solution (10 μg/ml amphotericin B, 1 mg/ml streptomycin, 0.04 mg/ml gentamycin and 0.1 mg/ml carbenicillin) in a six-well plate (Corning, New York, USA) with constantly gentle shaking for 3 h at 28 °C. Unsheathment of L3s were performed with sodium hypochlorite as described previously [28]. The exsheathed L3s were washed in PBS supplemented with the antibiotic-antimycotic solution mentioned above in a 6-well plate and were kept in gentle shaking for 6 h at 37 °C. Fresh sterilization fluids were changed hourly. After sterilization, the larvae were washed thoroughly several times in PBS to remove residual antibiotics.

The sterilized L1s were incubated in RPMI 1640 medium (25 mM HEPES, 1% fetal bovine serum, 10 μg/ml streptomycin and 10 U/ml penicillin), supplemented with 0 (control), 20, or 100 μM hemin chloride (Sigma-Aldrich) for 24 h at 28 °C. Simultaneously, the sterilized exsheathed L3s were cultured in RPMI 1640 medium (25 mM HEPES, 5 mM glutamine, 10 μg/ml streptomycin, 10 U/ml penicillin) containing the same concentrations of hemin chloride as above for 24 h at 37 °C. The stock solutions of hemin chloride were made by dissolving it in 300 mM ammonium hydroxide and the pH of the solution was adjusted to 8.0 followed by filter sterilization. After incubation, the larvae were harvested separately, washed three times in PBS and centrifuged at 1000×*g* for 5 min. The larvae were stored at − 80 °C for at least 2 days before use.

### Detection of Hc-HRG-2-heme complex

Twenty μM purified rHc-HRG-2 prepared in a binding buffer (250 mM Tris/HCl, pH 8.0, 5 mM EDTA and 10% glycerol) was incubated with equal concentration of heme at room temperature for 1 h. Staining with 3, 3’, 5, 5’-tetramethylbenzidine (TMB) was performed as described previously [29, 30]. In brief, the Hc-HRG-2-heme complex was separated by electrophoresis on 15% native-PAGE gels. The gels were stained with 15 ml of 2 mg/ml TMB (Sigma-Aldrich) dissolved in methanol and 35 ml of 0.5 M sodium acetate trihydrate (pH 5.0) for 5 min. Afterwards, 300 μl of 30% hydrogen peroxide (H_2_O_2_) were added into the mixture. Development of blue bands indicated the protein-heme complex. All these procedures were performed in dark. The loaded proteins were identified by Coomassie blue staining in a duplicate with identical treatment. The myoglobin prepared from equine heart (Sigma-Aldrich) was used as a positive control and the purified rHc-HRG-2 (20 μM) alone served as a negative control.

Recombinant Hc-HRG-2 proteins were purified by affinity chromatography followed by dialysis in PBS (pH 7.4) for 36 h with change of fresh PBS every 12 h. Afterwards, the proteins were concentrated using Amicon Ultra-15 centrifugal filters (Thermo Fisher Scientific, Shanghai, China). Protein concentration was determined using the Bradford Protein Quantitation Kit (Fude Biological Technology) and the protein solution was stored at − 80 °C before use.

Measurement of heme binding by rHc-HRG-2 was based on the heme titrations described earlier [31] and had been optimized in our laboratory. The assay was performed in PBS (pH 7.4) with different concentrations of heme. Stock solution of 10 mM hemin chloride (Sigma-Aldrich) was prepared in dimethyl sulfoxide (DMSO). The blanks contained only PBS, and the samples contained 5 μM proteins in reaction buffer. Heme was added to both blanks and samples to a final concentration of 2, 4, 6, 8, 10, 12 or 14 μM. The reaction took place at room temperature with constant rocking for 2 h before measured in a microplate reader. Heme binding was quantified based on a differential absorption peak at 414 nm. The ΔA_414 nm_ *versus* heme concentration data were plotted and analyzed using GraghPad Prism 6.0.

### GST activity measurement and inhibition studies

GSTs have the ability to catalyze the conjugation reaction between reduced glutathione (GSH) (Sangon Biotech) and CDNB (Sigma-Aldrich) to form a special product with absorption peaks at 340 nm. So, monitoring the change in absorbance at 340 nm over a certain period of time (Extinction coefficient Δε = 9.6 mmol^−1^cm^−1^) can be used to calculate the enzyme activity of GST.

The GST activity of Hc-HRG-2 was performed as described previously [32]. In brief, rHC-HRG-2 was purified and dialyzed in 0.1 M K_2_HPO_4_/KH_2_PO_4_ buffer (pH 6.5). The assays were conducted in 0.1 M K_2_HPO_4_/KH_2_PO_4_ buffer (pH 6.5) with different concentrations of CDNB. The reaction system was 200 μl with 1 mM GSH, 10 μM rHC-HRG-2 and various concentrations of CDNB at 0, 0.3, 0.5, 1, 1.5 or 2 μM. Each reaction was performed in triplicate and a complete assay mixture without rHC-HRG-2 was used as a negative control. The absorbance was measured in a microplate reader after 10 min of reaction time. The specific activity was calculated based on the Beer-Lambert law:

Specific activity (μmol/min/mg) = (ΔA_340_ × V)/(ε × T × L × E)

where ΔA340 is the change of absorbance value at 340 nm, V is the reaction volume (200 μl), ε is the extinction coefficient (9.6 mmol^−1^cm^−1^), T is the reaction time (10 minutes), L is the optical path (0.625cm); E: Enzyme quality (0.1 mg). The V_max_ (maximum velocity extrapolated to infinite substrate concentration) and K_m_ (substrate concentration that causes half-maximal enzyme velocity) were determined using GraghPad Prism 6.0.

For the inhibition assay of GST enzymatic activity, the experimental group contained 10 μM rHc-HRG-2 and 10 μM heme in 0.1 M K_2_HPO_4_/KH_2_PO_4_ buffer (pH 6.5), was incubated at room temperature for 2 h before measurement. The positive control contained 10 μM rHc-HRG-2 alone without heme and a reaction without rHC-HRG-2 was a negative control. Each sample was tested in triplicate. The absorbance at 340 nm was detected in microplate reader within 10 min. The ΔA_340_ under 10 min represented the GST enzymatic activity. The data were analyzed with GraghPad Prism 6.0.

## Results

### *Hc-hrg-2* gene structure

**Fig. 1:**
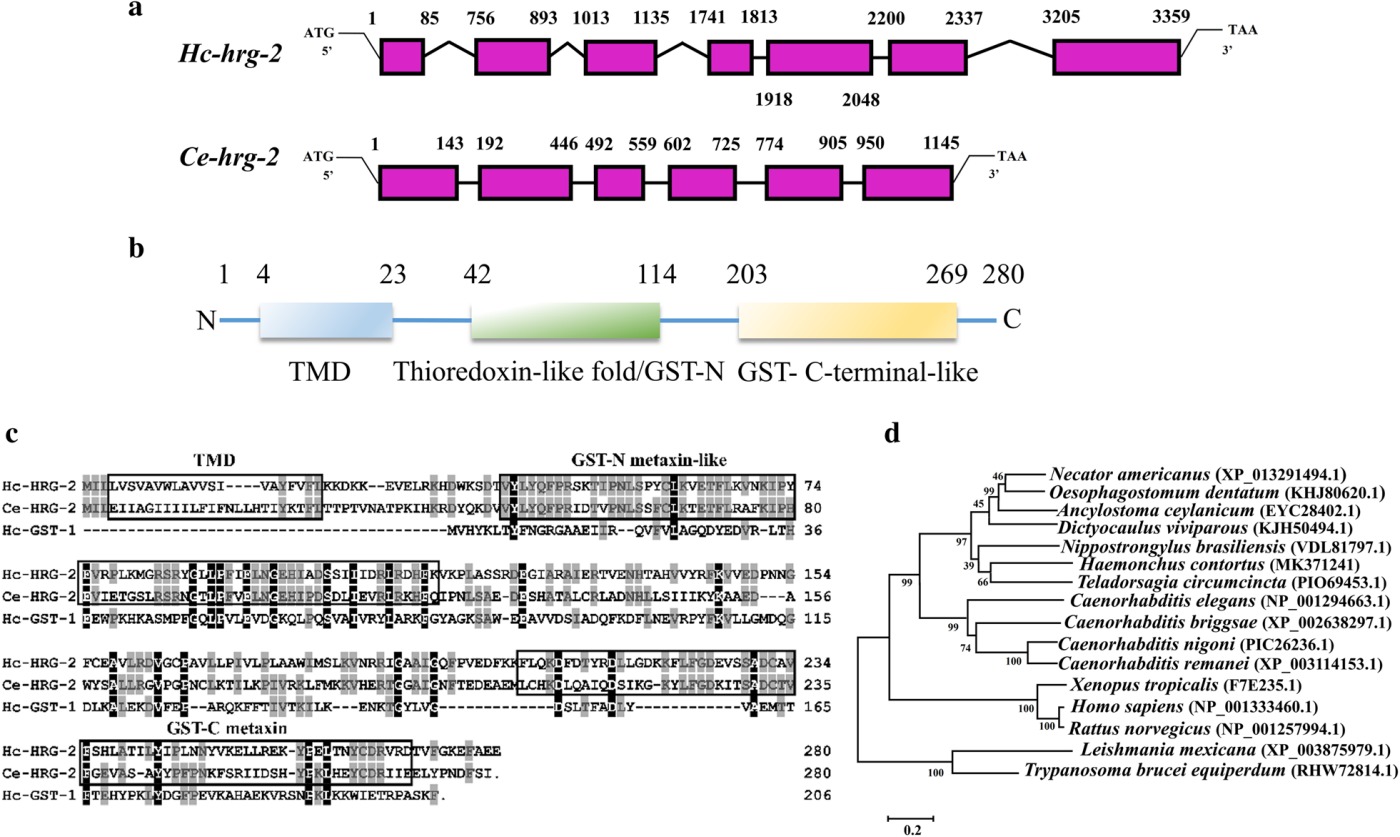
Sequence analysis of *Hc-hrg-2* in *H. contortus.* **a** The *hrg-2* genes of *H. contortus* and *C. elegans*. Pink boxes represent exons. The numbers above the pink boxes represent the positions in the genomes. **b** The putative domains of Hc-HRG-2. The transmembrane domain (TMD) was predicted by the TMHMM 2.0. The thioredoxin N-terminal domain-like (GST-N) and a glutathione S-transferase C-terminal domain-like (GST-C) folds were predicted using InterPro. **c** Multiple sequence alignment of Hc-HRG-2 with Ce-HRG-2 (WormBase ID: WBGene00010473) and Hc-GST-1 (GenBank: AF281663) by Clustal W method (Lasergene MegAlign software). **d** Phylogenetic analysis of HRG-2 sequences. Aligned protein sequences were subjected to phylogenetic analysis using the MEGA 5.1 software

A BLAST search using *Ce-hrg-2* (WormBase ID: WBGene00010473) as a query led to identification of a single candidate in *H. contortus*, named *H. contortus* heme-responsive gene-2 (*Hc-hrg-2*) (GenBank: MK371241). The *Hc-hrg-2* gene was 3359 bp in length and composed of 7 exons separated by 6 introns (Fig. 1a). The gene structure was more complicated than that of *Ce-hrg-2* (Fig. 1a). The ORF of *Hc-hrg-2* was 843 bp and encoded 280 amino acids with a predicted molecular weight of 32.3 kDa (Fig. 1b). The annotated Hc-HRG-2 protein shared 50–65% similarity to homologs of *Ancylostoma ceylanicum*, *Dictyocaulus viviparous*, *Necator americanus* and *Oesophagostomum dentatum* and 37.5% similarity to that of *C. elegans* (data not shown). Two signature domains, a thioredoxin-like (GST-N) at position 42–114 and a glutathione S-transferase-C-terminal domain-like (GST-C) at position 203–269 (Fig. 1c) were predicted. However, Hc-HRG-2 shared only 19.4% similarity to Hc-GST-1 (GenBank: AF281663) (Fig. 1c). In addition, a single transmembrane domain at position 4–23 was predicted at the N terminus (Fig. 1c). Phylogenetic analysis showed that Hc-HRG-2 and Ce-HRG-2 were in the same clade, and that Hc-HRG-2 was highly conserved in parasitic nematodes (Fig. 1d). A topological structure prediction showed that Hc-HRG-2 had the closest similarity to 4KF9 in PDB library which is a glutathione transferase family member from *Ralstonia solanacearum* (https://www.rcsb.org/structure/4KF9) (data not shown). It was further predicted that Hc-HRG-2 was capable of binding to GSH. Based on these bioinformatics data, we postulated that Hc-HRG-2 is likely involved in heme transport in addition to GST enzymatic activity.

### mRNA transcription of *Hc-hrg-2* peaks at L3 during the *H. contortus* life-cycle

**Fig. 2:**
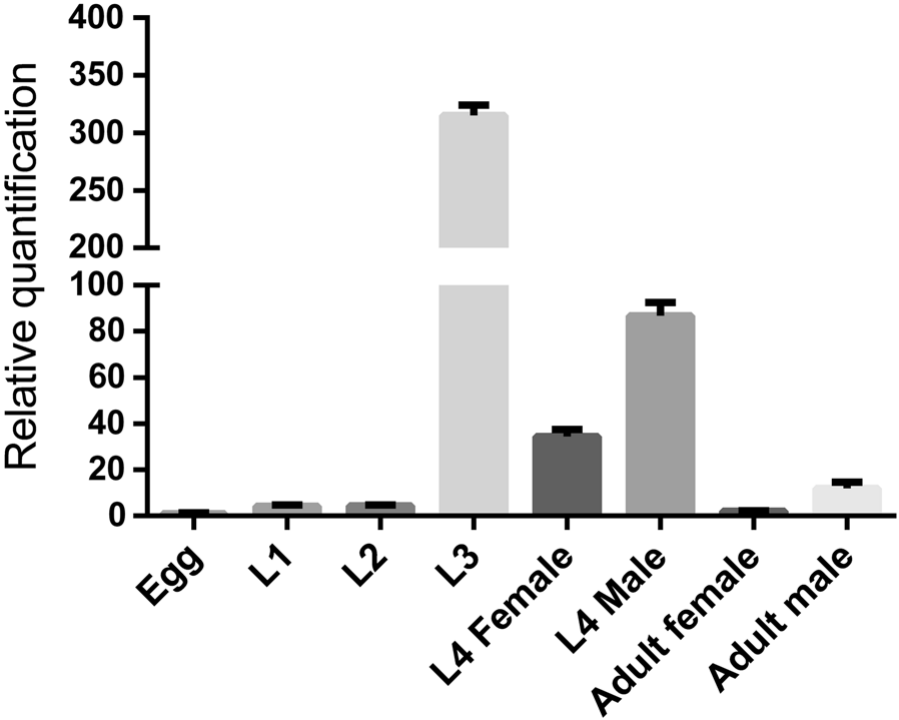
Transcriptional levels of *Hc-hrg-2* at different developmental stages of *H. contortus.* The abundance of *Hc-hrg-2* transcripts were quantified by qRT-PCR. Relative quantification were derived by normalizing the cycle threshold values to *Hc-actin-1* and then to the control life stage of egg using 2^−ΔΔCT^ methods

The transcription levels of *Hc-hrg-2* in different developmental stages of *H. contortus* was performed by qRT-PCR. The highest level of the *Hc-hrg-2* transcript reached approximately 325 times that of *actin-1* at the L3 stage (Fig. 2). In comparison, the embryo, L1s and L2s showed the lowest transcription of *Hc-hrg-2*, which was only about 1% of that of L3s (Fig. 2). The transcription of *Hc-hrg-2* in the blood-feeding stages (female L4s, male L4s, female adults and male adults) dropped dramatically to 4–30% of that of L3s.

### *Hc-hrg-2* is induced under high concentration of heme in *H. contortus*

**Fig. 3:**
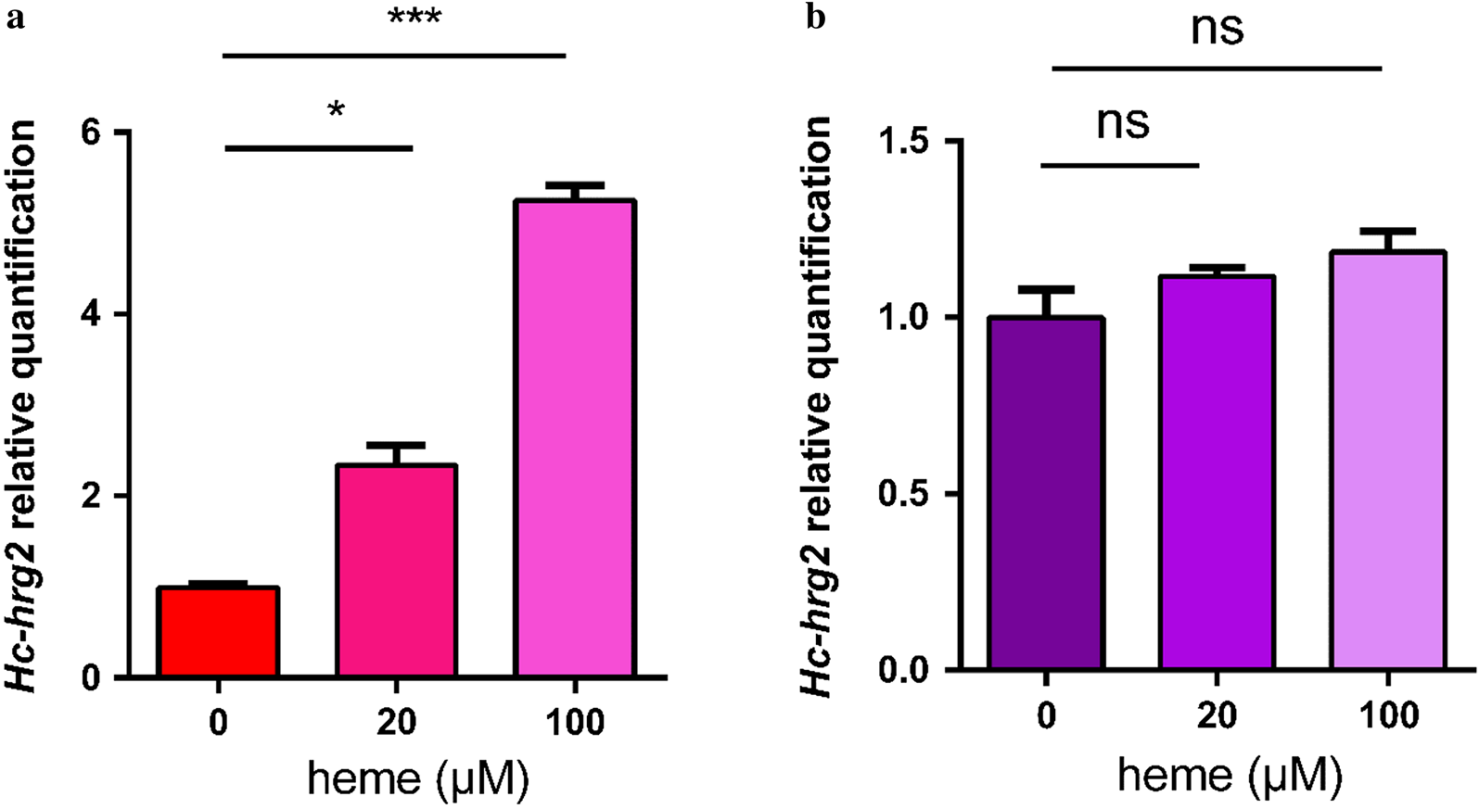
*Hc-hrg-2* is a heme-responsive gene. The transcription of *Hc-hrg-2* was estimated by qRT-PCR from total RNA obtained from L1s and exsheathed L3s grown at the indicated concentrations of heme. **a** The abundance of *Hc-hrg-2* mRNA in exsheathed L3s.*P < 0.05, ***P < 0.001. **b** The abundance of *Hc-hrg-2* mRNA in L1s. ns: no statistical significance. The results are the means ± SE of 3 independent experiments. The gene expression level was normalized to *Hc-actin-1* and the levels of controls at 0 μM heme were set at an arbitrary one unit

To determine whether Hc-HRG-2 indeed responds to heme, we investigated the transcription of *Hc-hrg-2* in the L1s and the exsheathed L3s of *H. contortus* under various concentrations of exogenous heme at 0, 20 or 100 μM. The transcription of *Hc-hrg-2* in exsheathed L3s cultured at 100 μM heme was five times greater than that of worms cultured in the absence of heme (0 μM) (Student’s t-test: *t*_(2)_ = 9.563, *P* = 0.0108; *t*_(2)_ = 40.01, *P* = 0.0006) (Fig. 3a). In contrast, there was no significant difference between samples treated with various concentrations of heme in L1s (Fig. 3b). These data revealed that the transcription of *Hc-hrg-2* was upregulated in the presence of heme in a dose-dependent manner in exsheathed L3s of *H. contortus*, but not in L1s.

### TMD of Hc-HRG-2 is required for its targeting to the endoplasmic reticulum (ER)

**Fig. 4:**
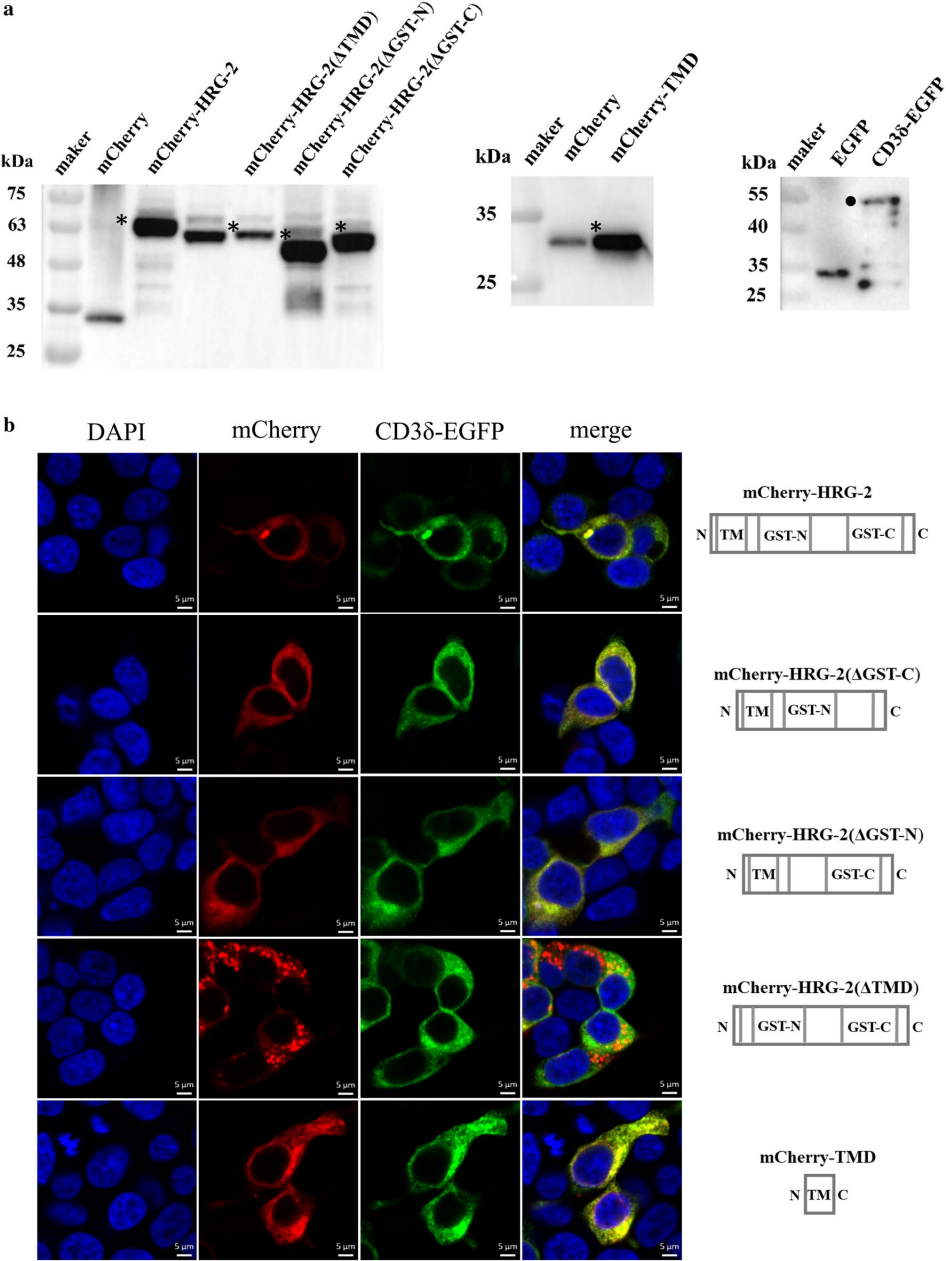
Hc-HRG-2 is a transmembrane protein targeted to the endoplasmic reticulum. **a** Western blot analysis of Hc-HRG-2, truncated Hc-HRG-2 and CD3δ-EGFP transiently expressed in HEK293T cells. The whole cell lysates of transfected cells were subjected to SDS-PAGE and Western blot using anti-mCherry or anti-GFP antibodies. Asterisks and circles indicate the target protein. **b** Confocal microscopy of Hc-HRG-2 and CD3δ-EGFP co-expressed in HEK293T cells. The full length and truncated Hc-HRG-2 are shown on the right of each row. *Scale-bars*: 10 μm

Ce-HRG-2 localizes to the ER in *C. elegans* [23]. Based on the high homology of *Hc-hrg-2* with *Ce-hrg-2*, we generated the Hc-HRG-2 with a mCherry fusion at the N-terminus and co-expressed with the ER marker CD3δ-EGFP in the 293T cells to determine the subcellular distribution of HRG-2 in *H. contortus*. Western blot analysis of lysates from HEK293 cells transfected with either Hc-HRG2 or truncated Hc-HRG-2 showed that the tagged Hc-HRG-2 migrated at the expected molecular weight (Fig. 4a). Fluorescence microscopy studies showed that Hc-HRG-2 was co-localized with the ER marker (Fig. 4b, top row), consistent with the localization of Ce-HRG-2. To identify the functional domain that determines cell localization, we generated truncated Hc-HRG-2 constructs and expressed them in 293T cells. The truncated proteins without the GST-N or GST-C domain were still localized in the ER (Fig. 4b, second and third rows). However, the proteins without TMD had a punctate distribution (Fig. 4b, forth row). By contrast, fusion the TMD to mCherry directly resulted in ER localization (Fig. 4b, bottom row). These results demonstrate that the N-terminal TMD is required for targeting Hc-HRG-2 to ER membrane.

### Hc-HRG-2 is located in the hypodermal tissues of adult *H. contortus*

**Fig. 5:**
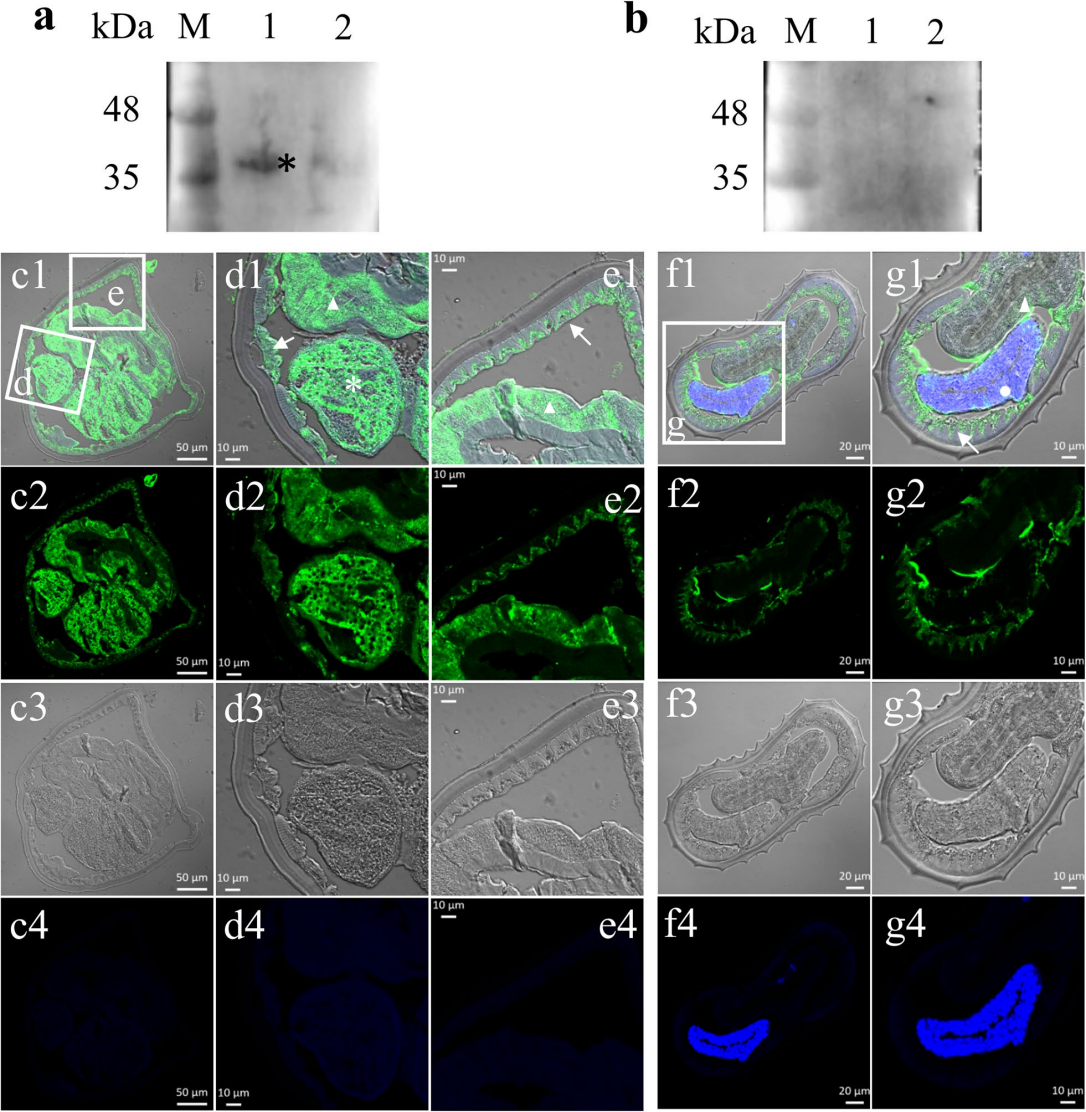
Immunofluorescence analysis of Hc-HRG-2 in adult *H. contortus*. Neutral Hc-HRG-2 was detected using mouse anti-Hc-HRG-2 polyclonal antibody and fluorophore-conjugated anti-mouse IgG antibodies in the paraffin section of worms. **a** Mouse anti-Hc-HRG-2 polyclonal antibody recognized the natural protein of adult *H. contortus* (black asterisks, target band). **b** A negative control using pre-immune serum as primary antibody. Lane 1: PBS-soluble natural protein; Lane 2: urea-soluble natural protein. **c** Cross-section of a female adult (*scale-bars*: 50 μm). **f** Cross-section of a male adult (*scale-bars*: 20 μm). Merge, GFP, DIC and DAPI labeled as 1, 2, 3 and 4, respectively. **d, e** and **g** represent the magnified parts of the sections (*scale-bars*: 10 μm). *Key*: triangles, intestine; asterisks and circles, gonad; white arrows, body hypodermal tissues

To determine the tissue localization of Hc-HRG-2, we performed immunofluorescence histochemistry on paraffin sections of adult *H. contortus* using the polyclonal antibody to rHc-HRG-2. The recombinant protein Hc-HRG-2 was highly expressed in *E. coli* BL21 and purified by Ni-NTA agarose column as shown by SDS-PAGE (Additional file 1: Figure S1a, b). Western bolt using the polyclonal antibody generated against rHc-HRG-2 detected a single band of approximately 38 kDa among whole cellular lysates of adult worms, which confirmed the specificity of these antibodies to the endogenous wild-type Hc-HRG-2 protein (Fig. 5a, b). Immunofluorescence histochemistry assays showed that the body hypodermal tissues of both female and male worms had high levels of GFP signal, which indicated that HRG-2 was mainly located in the hypodermal tissues (Fig. 5c–g). It is worth noting that Hc-HRG-2 was also expressed in the intestines and gonads of female worms, suggesting that Hc-HRG-2 may also be involved in nutrient absorption and embryo development (Fig. 5c–e).

### Binding of Hc-HRG-2 to heme exhibits peroxidase activity

**Fig. 6:**
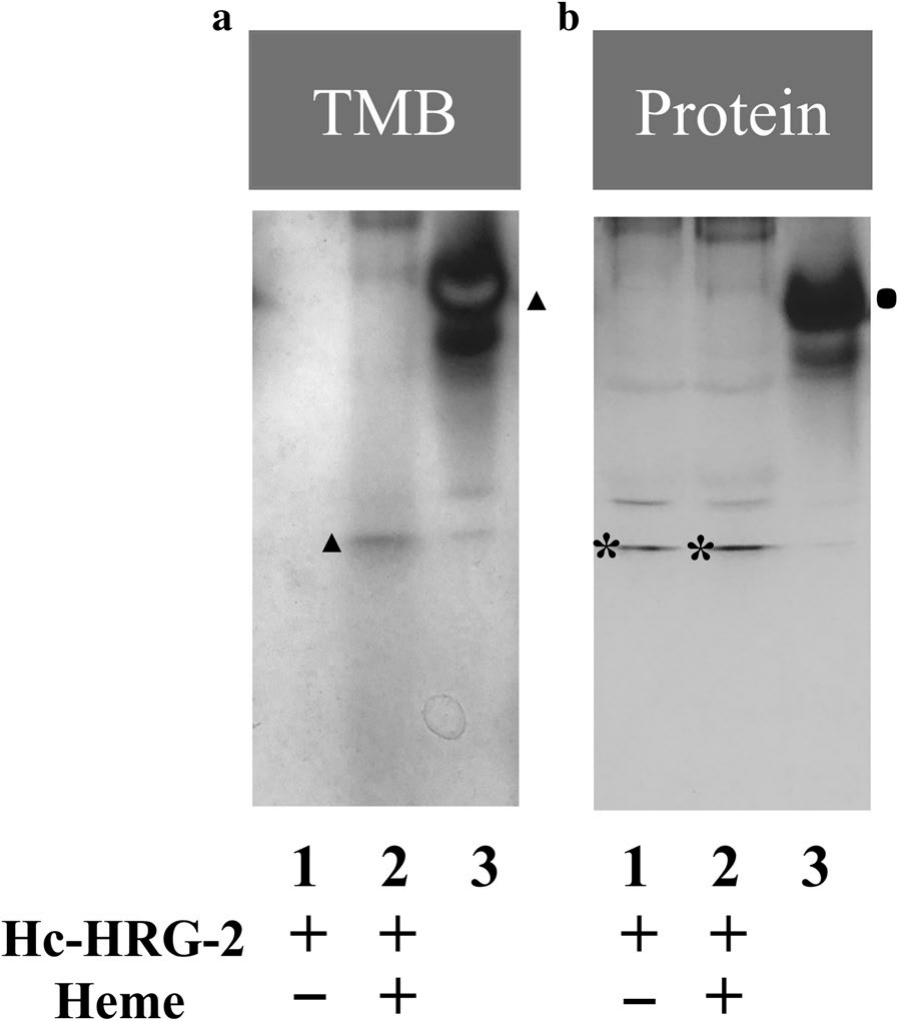
The rHc-HRG-2-heme complex exhibits peroxidase activity. Purified proteins were incubated with heme at 37 °C for 2 h. Thereafter, the formed complexes were resolved by native PAGE. **a** The presence of heme revealed by TMB (triangle: target band). **b** The presence of protein by Coomassie blue staining (asterisks: target bands for rHc-HRG-2; dot: target band for equine heart myoglobin). Lane 1: rHc-HRG-2 only; Lane 2: rHc-HRG-2 plus heme; Lane 3: the positive control, equine heart myoglobin

One important characteristic of hemoproteins is their peroxidase activity that can be detected in the presence of H_2_O_2_ and a hydrogen donor such as TMB in polyacrylamide gels [30]. To test whether Hc-HRG-2 interacts directly with heme, we used the TMB-H_2_O_2_ as a stain to detect the peroxidase activity of rHc-HRG-2-heme complex. Staining results showed that there was a single blue band in the rHc-HRG-2-heme complex lane but not in the rHc-HRG-2 lane, which indicated that rHc-HRG-2 interacted with heme directly and this heme-protein complex had peroxidase activity (Fig. 6).

### Hc-HRG-2 with a GST activity is more like a heme binding protein

**Fig. 7:**
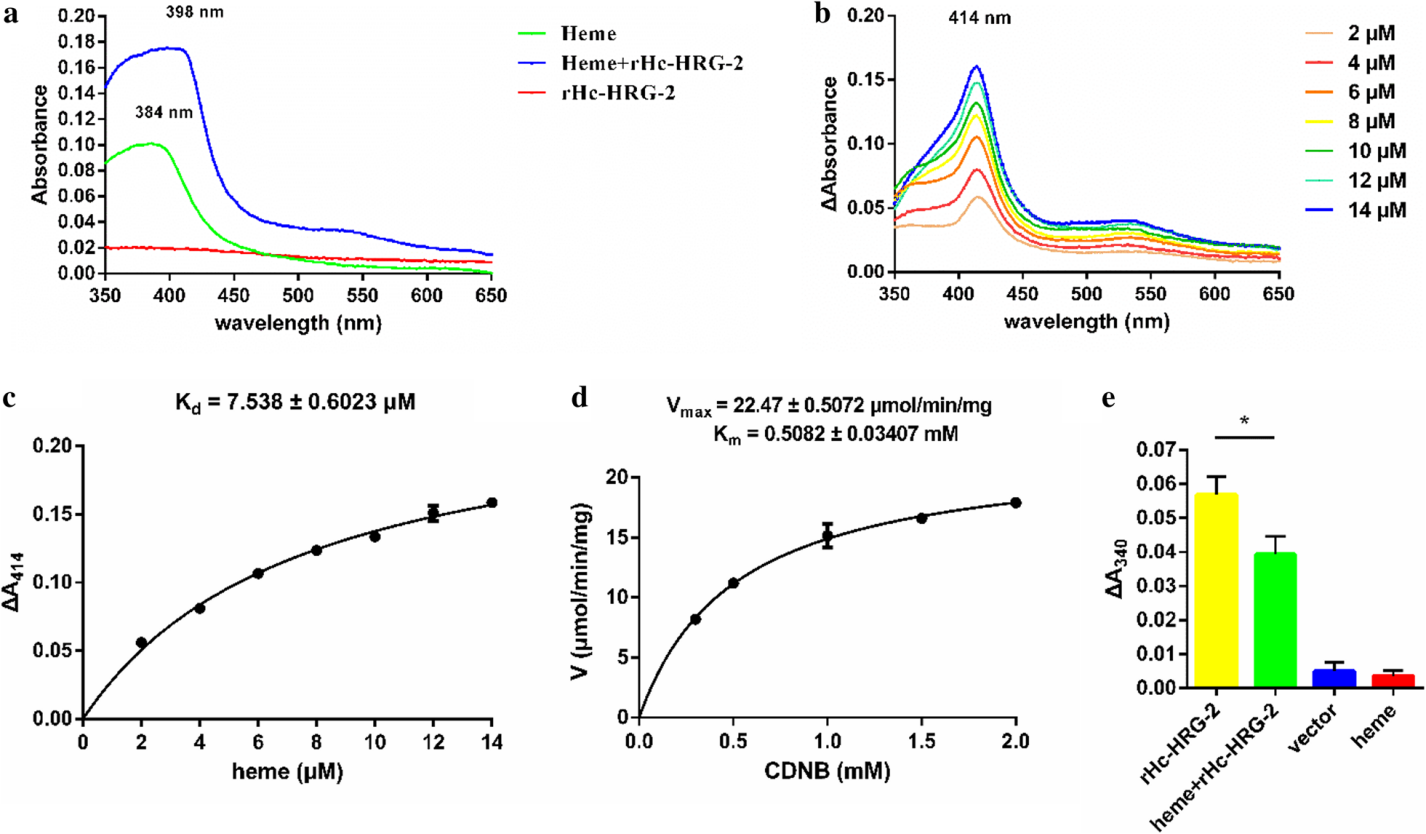
Heme-binding and GST enzymatic activities of rHc-HRG2. **a** UV-visible absorption spectra recorded in the presence of 5 μM rHc-HRG2 and 6 μM heme. **b** Difference absorption spectra recorded from the heme titration experiment using 5 μM rHc-HRG2 and various concentrations of heme ranging from 2 to 14 μM. **c** Heme-binding curve generated from the difference absorption spectra in panel **b** by plotting ∆A_414_ *versus* the heme concentration. The results represent the mean ± SE of three independent experiments. **d** The GST activity of rHc-HRG-2 (10 μM), representing the mean ± SE of triplicates. **e** Inhibition of GST activity by the binding of heme (10 μM) to rHc-HRG-2 (10 μM). *P < 0.05

The peroxidase activity as a heme-rHc-HRG-2 complex shown above (Fig. 6) suggested that Hc-HRG-2 binds heme. To test this hypothesis, a heme titration was performed, and UV-visible absorption spectra were recorded after titration of proteins (5 μM) with heme (Fig. 7a, b). The difference spectra exhibited absorbance changes in the Soret region at 414 nm (Fig. 7b). By plotting the change in absorbance at 414 nm *versus* heme concentration, a typical hyperbola was obtained, which indicated saturation binding (Fig. 7c.). The K_d_ value was 7.538 ± 0.6023 μM (mean ± standard error, SE) which was in good agreement with previously published data on a heme binding protein Tfo of *Tannerella forsythia* [33]. These data indicate that rHc-HRG-2 binds to heme with a high affinity *in vitro*.

We then investigated whether Hc-HRG-2 served in the cellular detoxification by mediating endogenous or xenobiotic compounds to conjugate with GSH to increase their water solubility and excretion [34, 35]. The GST enzymatic activity of rHc-HRG-2 catalyzing the conjugation of GSH to the model substrate CDNB was estimated to be 22.47 ± 0.5072 μmol/min/mg (mean ± SE), indicating that rHc-HRG-2 functions as a glutathione transferase *in vitro* (Fig. 7d).

To further study whether heme influences its GST enzymatic activity, rHc-HRG-2 was pre-incubated with an equivalent molar concentration of heme prior to the reaction. Its enzyme activity was reduced by 31% with statistically significant differences between the experimental group and the positive control group (Student’s t-test: *t*_(2)_ = 4.678, *P* = 0.0428) (Fig. 7e), indicating that heme and CDNB may share the same binding site on Hc-HRG-2.

## Discussion

Red blood cells account for about half of the blood volume and are rich in hemoglobin (150 mg/ml). Their effective lysis and catabolism are the core requirements for the blood-feeding parasites [36]. The hydrolysis of hemoglobin yields large amounts of toxic free heme. All blood-feeding parasites, including *H. contortus*, are equipped with molecular mechanisms for the efficient disposal and detoxification of their heme surplus [37]. However, these mechanisms remain elusive for many parasite species.

In the present study, we first identified the *Hc-hrg-2* gene, which was highly upregulated when worms were exposed to high concentrations of heme. Further, Hc-HRG-2 was mainly expressed in the hypodermal tissues of *H. contortus* and localized in the endoplasmic reticulum in transfected mammalian cells. We demonstrated that Hc-HRG-2 was a heme-binding protein with GST enzymatic activity. The enzymatic inhibition assay revealed that heme had a significant effect on the enzyme activity of Hc-HRG-2 with the model substrate CDNB.

The mRNA profile showed that the highest level of the *Hc-hrg-2* transcript was in L3s and the lowest level in L1s, indicating that heme is only able to induce the responsiveness of *Hc-hrg-2* in the L3s (Figs. 2, 3). Considering that the exogenous heme is mainly absorbed through the intestine in the L1s and L2s, there is another possibility that some heme transporters in the intestine are involved in the regulation of heme homeostasis directly [38]. The low transcription and expression pattern of *Hc-hrg-2* in the adult stage indicates that the gene may play less significant role in heme response at this stage (Fig. 2). In the blood-feeding stages, the intestine of *H. contortus* encounters large mass of toxic free heme hydrolyzed from host Hb directly [39]. A variety of heme detoxification mechanisms have been proved to exist in blood-feeding nematodes, including the breakdown of heme into Fe and less reactive intermediates, the containment of heme through a physical barrier, and the conversion of heme to an inert crystal structure [13]. Obviously, heme metabolism in the blood-feeding stage is quite different to that in the free-living stage. The transcription levels of *Hc-hrg-2* reached its peak in L3 and L4 suggests that Hc-HRG-2 may be also related to the regulation of the pivotal transition from the free-living stages to the blood-feeding stages (Fig. 2). It should be noted that *Ce-hrg-2* is upregulated in a heme-deficient environment [23], which is contrary to the result for *Hc-hrg-2*. This may be due to the difference in the living environment where *C. elegans* is free-living throughout its entire life-cycle and relies on a small amount of environmental heme [40–42].

It should be logical to perform the immunolocalization in L3s where *Hc-hrg-2* is transcripted with the highest level rather than adults. However, we have failed to locate Hc-HRG-2 in the L3s due to technical limitations. Instead, immunofluorescence histochemistry assays in adult *H. contortus* successfully and clearly indicated that Hc-HRG-2 is mainly located in the hypodermal tissues (Fig. 5).

Compared with Ce-HRG-4, a multi-transmembrane permease mediating heme uptake at the plasma membrane [38], Hc-HRG-2 contains only one transmembrane domain (Fig. 1). It seems that Hc-HRG-2 is not a heme transporter. Heme-binding activities have been demonstrated for GSTs from many parasites, such as *Ancylostoma caninum* and *Necator americanus* [22, 43]. It seems like that Hc-HRG-2 binds to heme depending on its GST-like domain. Certainly, this GST-like hypothesis requires more experimental data to be tested. Additionally, the expression of Hc-HRG-2 in the ER further indicated that Hc-HRG-2 might mediate heme delivery to membrane-anchored or luminal hemoproteins. The blood-feeding parasites have evolved various detoxification mechanisms to cope with the heme toxicity, including the breakdown of heme into molecular iron and less reactive intermediates [13]. Since Hc-HRG-2 has a thioredoxin-like fold, we speculate that it may also act as a membrane-associated oxidoreductase, which may facilitate iron uptake [44, 45].

The GST enzyme activity of Hc-HRG-2 was significantly inhibited by its binding to heme (Fig. 7e) with the model substrate CDNB. Further research is needed to determine whether heme and CDNB share the same binding site, such as measuring the 50% inhibitory concentration of heme for the GSH-CDNB conjugation catalyzed by Hc-HRG-2. A previous study has shown that GSH can produce free radicals to cleave heme at the porphyrin ring in the presence of oxygen [46]. It is possible that Hc-HRG-2 acts by binding heme and presenting it to GSH. Although much of the attention on vaccination against hookworms and schistosome has focused on GSTs [47], it is also necessary to explore whether Hc-HRG-2 could be a vaccine candidate.

## Conclusions

In this study, we showed that *Hc-hrg-2* was highly upregulated under high concentrations of heme and that Hc-HRG-2 was mainly expressed in the hypodermal tissues of *H. contortus* and localized in the endoplasmic reticulum in a mammalian cell line. Our functional studies indicated Hc-HRG-2 is a heme-binding protein with a GST enzymatic activity and that heme has a significant effect on this enzyme activity. In summary, we demonstrated that *Hc-hrg-2* is a heme-responsive gene and engaged in heme homeostasis regulation in hypodermal tissues in the free-living stages of *H. contortus*.

## Supplementary information

**Additional file 1: Figure S1. a** pET32a-*Hc-hrg-2* transformed into *E. coli* (BL21) and induced in 37 °C in different temperature. Lane M: marker; Lane 1: 0 h; Lane 2: 2 h; Lane 3: 4 h; Lane 4: 6 h; Lane 5: 8 h; Lane C: control (pET32a empty). **b** The recombinant protein Hc-HRG-2 was purified by Ni-NTA agarose column. Lane M: marker; Lanes 1–2: 60 mM imidazole-eluted protein; Lanes 3–11: 250 mM imidazole-eluted protein. Arrows indicate the target bands of rHc-HRG-2.

## Data Availability

All data supporting the conclusions of this article are included within the article and its additional file. The sequence of *Hc-hrg-2* had been submitted to the GenBank database under the accession number MK371241.

## Abbreviations

ALAD: δ-aminolevulinic acid dehydratase
CDNB: 1-chloro-2, 4-dinitrobenzene
DAPI: 4’, 6-diamidino-2-phenylindole
DMSO: dimethyl sulfoxide
EDTA: ethylene diamine tetraacetic acid
ELISA: enzyme-linked immunosorbent assay
ER: endoplasmic reticulum
FC: ferrochelatase
GSH: glutathione
GST: glutathione S-transferase
GST-C: glutathione S-transferase C-terminal domain-like
GST-N: thioredoxin N-terminal domain-like
hrg: heme-responsive gene
iL3s: infective third-stage larvae
IPTG: isopropy beta-D-thiogalactopyranoside
I-TASSER: Iterative threading ASSEmbly Refinement
L1s: first-stage larvae
L2s: second-stage larvae
L3s: third-stage larvae
L4s: fourth-stage larvae
Native-PAGE: non-denaturing polyacrylamide gel electrophoresis
NJ: neighbor-joining
ORF: open reading frame
PBGD: porphobilinogen deaminase
PBS: phosphate-buffered saline
PMSF: phenylmethanesulfonyl fluoride
qRT-PCR: quantitative reverse transcription-PCR
RBC: red blood cell
rHc-HRG-2: recombinant Hc-HRG-2
SDS-PAGE: SDS-polyacrylamide gel electrophoresis
SE: standard error
TMB: 3, 3ʹ, 5, 5’-tetramethylbenzidine
TMD: transmembrane domain
ZJ strain: Zhejiang strain
