# Supplementary material for: Hc-hrg-2, a glutathione transferase gene, regulates heme homeostasis in the blood-feeding parasitic nematode Haemonchus contortus
Source: Parasit Vectors. 2020 Jan 29;13:40. doi: 10.1186/s13071-020-3911-z (PMC6988263; doi:10.1186/s13071-020-3911-z)
Supplement: Supplementary file 1 — Additional file 1: Figure S1. a pET32a-Hc-hrg-2 transformed into E. coli (BL21) and induced in 37 °C in different temperature. Lane M: marker; Lane 1: 0 h; Lane 2: 2 h; Lane 3: 4 h; Lane 4: 6 h; Lane 5: 8 h; Lane C: control (pET32a empty). b The recombinant protein Hc-HRG-2 was purified by Ni-NTA agarose column. Lane M: marker; Lanes 1–2: 60 mM imidazole-eluted protein; Lanes 3–11: 250 mM imidazole-eluted protein. Arrows indicate the target bands of rHc-HRG-2. [file 13071_2020_3911_MOESM1_ESM.pdf]

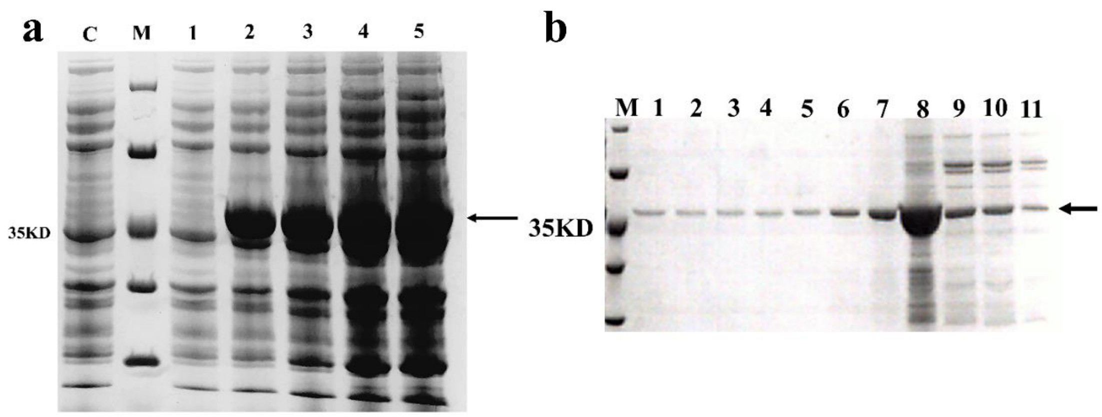

Additional file 1: Figure S1 a pET32a-*Hc-hrg-2* transformed into *E. coli* (BL21) and induced in 37 ℃ in different temperature. Lane M: Marker; Lane 1: 0 h; Lane 2: 2 h; 3: 4 h; Lane 4: 6 h; Lane 5: 8 h; Lane C: Control (pET32a empty). **b** The recombinant protein Hc-HRG-2 was purified by Ni-NTA agarose column. Lane M: Marker; lane 1-2: 60 mM imidazole-eluted protein; lane 3-11: 250 mM imidazole-eluted protein. Arrow: the target bands of rHc-HRG-2.
